# Supplementary material for: Polymorphisms in Pfkelch13 domains before and after the introduction of artemisinin-based combination therapy in Southwest Nigeria
Source: PLoS One. 2025 Mar 31;20(3):e0316479. doi: 10.1371/journal.pone.0316479 (PMC11957316; doi:10.1371/journal.pone.0316479)

Samples: 12024
Bases: 1076
Average spacing: 12

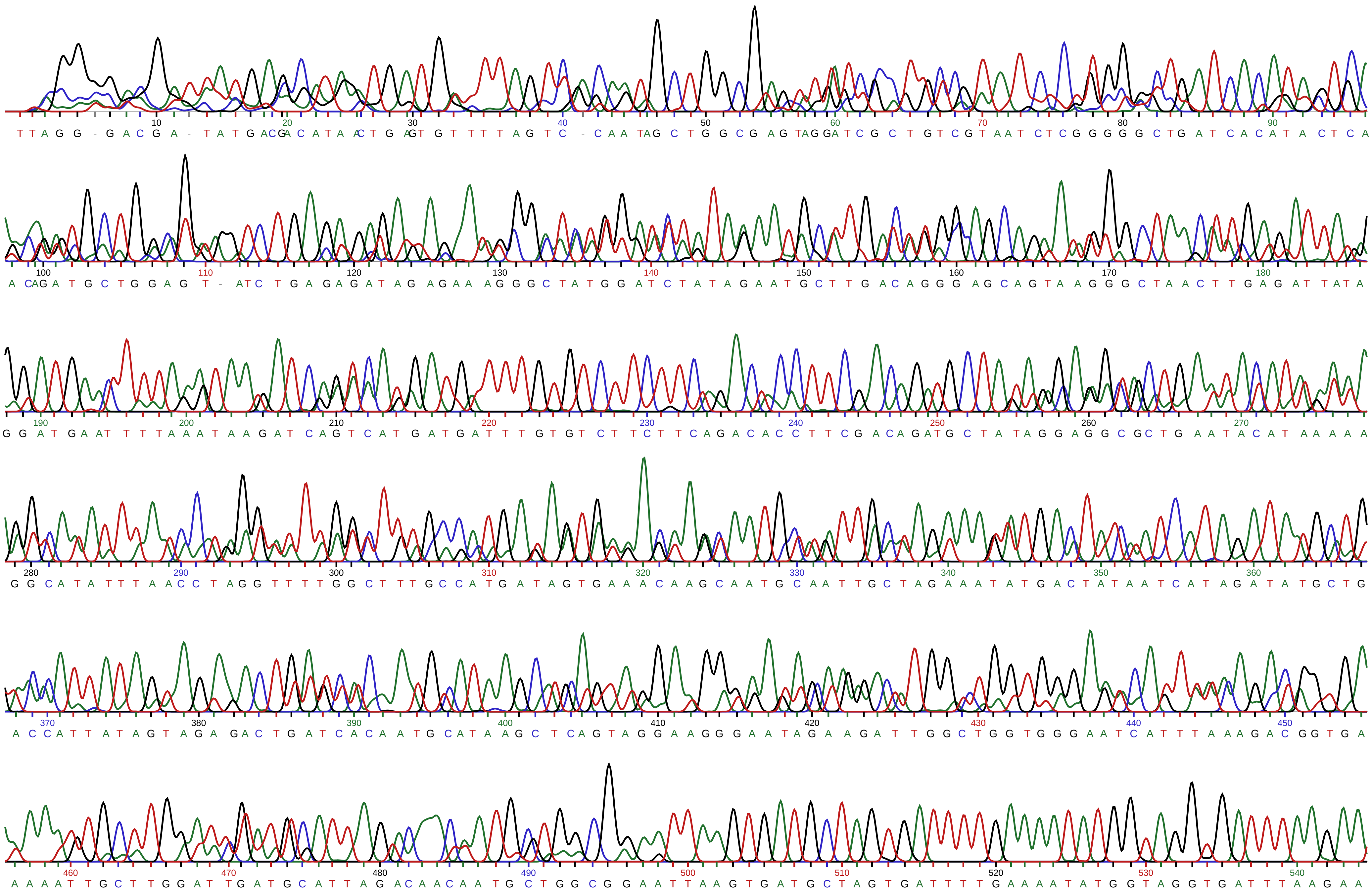

Samples: 12024
Bases: 1076
Average spacing: 12

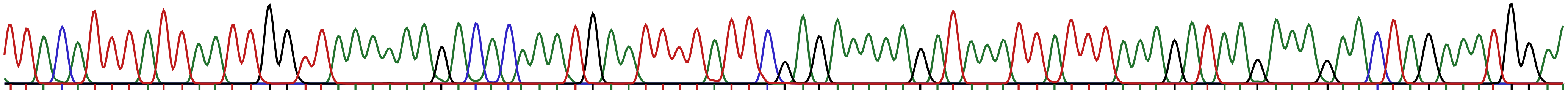

550 560 570 580 590 600 610 620 630

T T A C A T T T A T T A A T T G G T T A A A A A A G A C A C A A A T G A A T T T T A T T C G A G A A A A A G A T A A A T T A T T T A A A G A T A A G A A A G A A C T A G A A A T G G A A

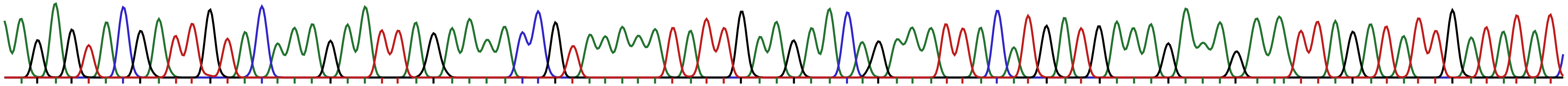

640 650 660 670 680 690 700 710 720

A G A G T A C G A T T G T A C A A A G A A T T A G A A A A C C G T A A A A A T A T T G A A G A A C A G A A A T T A C A T G A T G A A A G A A A G A AA T T A G A T A T T G A T AT AT

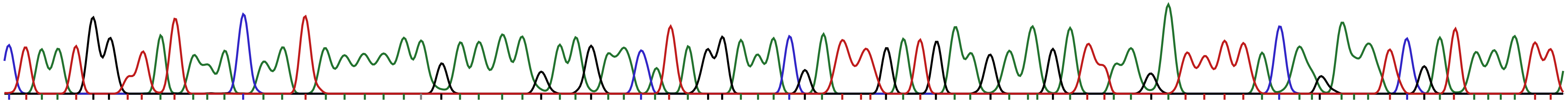

730 740 750 760 770 780 790 800 810

C T A A T G G T T A T A A A C A A T A A A A A - G A A A A G A A G A A C A T A GG A A A C G A T TT G A T G A A G A AA G A T TA A G A T T T T A C AAG A A A T C G A T A A A A T T

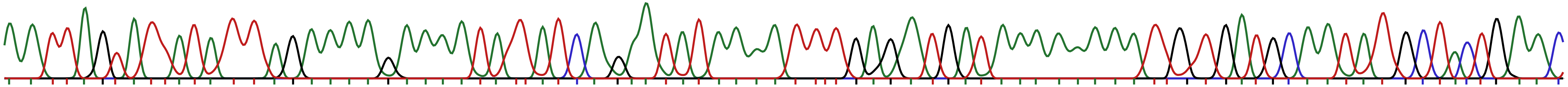

820 830 840 850 860 870 880 890 900

A A TT A GT A TT A T A T T A G A A A A G A A A A T A TT A T C A G A A T A T A A A A T TTT G A G A T G A T A A A A A A A A TT G T G A T G C A A T A T G C T A C T G A A C

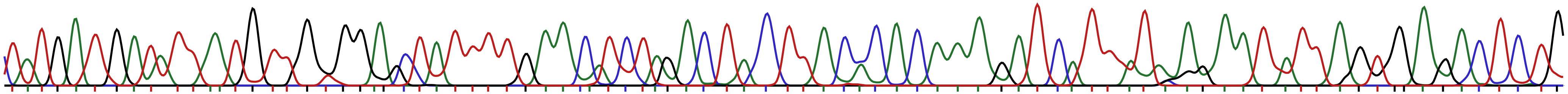

910 920 930 940 950 960 970 980 990

T A T G A T G A T T T AA T G T T G T G G AG C T A T T T T G A A CA T C T AG A C T A C T T A C A C A C A A A G A T C A T T A T A A G A A T A T T A G T GG A G A C T C T G

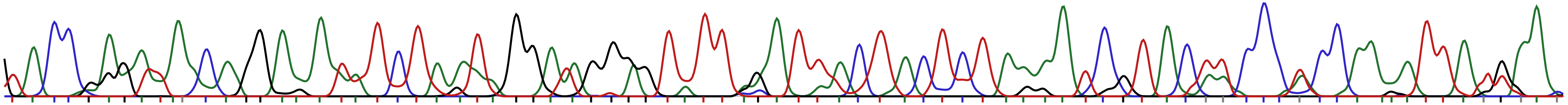

1000 1010 1020 1030 1040 1050 1060 1070

T A C C G A G A T A- C A G G A A A T A T C T A A T A G G A A G G G G T A T T A G A T T A C T A C T C T A A A A T C G T A C - - C C C - C C A AA A T T A - G - A -

Samples: 12024
Bases: 1076
Average spacing: 12

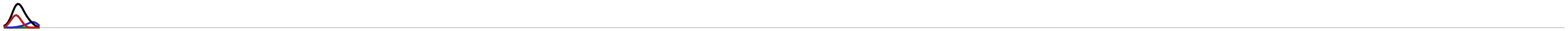

Supplement: Supporting information 2 — (ZIP) [file pone.0316479.s002.zip › 182KN2F_PREMIX_Plate_KELCH2_C05.pdf]
